# In vitro activity of chemicals and commercial products against *Saprolegnia parasitica* and *Saprolegnia delica* strains

**DOI:** 10.1111/jfd.12923

**Published:** 2018-12-10

**Authors:** Perla Tedesco, Maria Letizia Fioravanti, Roberta Galuppi

**Affiliations:** ^1^ Department of Veterinary Medical Sciences Alma Mater Studiorum University of Bologna Ozzano Emilia (BO) Italy

**Keywords:** in vitro test, minimum inhibitory concentration, minimum lethal concentration, *Saprolegnia* spp., treatment

## Abstract

Oomycetes of the genus *Saprolegnia* are responsible for severe economic losses in freshwater aquaculture. Following the ban of malachite green in food fish production, the demand for new treatments pushes towards the selection of more safe and environment‐friendly products. In the present work, in vitro activity of ten chemicals and three commercial products was tested on different strains of *Saprolegnia*, using malachite green as reference compound. The compounds were screened in agar and in water to assess the minimum inhibitory concentration (MIC) and the minimum lethal concentration (MLC), respectively. Two strains of *Saprolegnia parasitica* and one isolate of *Saprolegnia delica* were tested in triplicate per each concentration. Among tested chemicals, benzoic acid showed the lowest MIC (100 ppm) followed by acetic acid, iodoacetic acid and copper sulphate (250 ppm). Sodium percarbonate was not effective at any tested concentration. Among commercial products, Virkon^™^S was effective in inhibiting the growth of the mycelium (MIC = MLC = 1,000 ppm). Actidrox® and Detarox® AP showed MIC = 5,000 and 1,000 ppm, respectively, while MLCs were 10‐fold lower than MICs, possibly due to a higher activity of these products in water. Similarly, a higher effectiveness in water was observed also for iodoacetic acid.

## 1 INTRODUCTION

The genus *Saprolegnia* includes oomycetes that are widely distributed in the aquatic environment; most members of this genus are saprotrophs, however, some species can be pathogenic to fish (Van West, 2006). *Saprolegnia* infections are an economically relevant aspect of salmonid aquaculture, causing severe losses in the farmed stock, and are characterized by the presence of whitish or greyish cotton‐like tuft on eggs or over the skin and gills of fish, on which it may spread and cover more than 80% of the body surface (Van Den Berg, McLaggan, Diéguez‐Uribeondo, & Van West, 2013). The infection causes the destruction of the epidermis and hyphae can reach the underlying fish tissues (Pickering & Willoughby, 1982) and blood vessels (Hatai & Hoshiai, 1992), leading to impaired osmoregulation. Respiratory failure may also occur as a consequence of gill infection. Behavioural changes include lethargy and loss of equilibrium. If untreated, saprolegniosis may lead to death of the infected fish (Van West, 2006).

The apparent low efficacy of prophylactic measures and the scarcity of registered treatments for the control of saprolegniosis urge the screening of new molecules and products active against *Saprolegnia* spp.

At present, no alternative treatment with effectiveness comparable to malachite green (MG) has been found for *Saprolegnia* infections (Sudova, Machova, Svobodova, & Vesely, 2007). MG has long been used in aquaculture (Foster & Woodbury, 1936) as effective fungicide, and for the treatment of protozoan infections (*Ichtyophthirius* sp., Rahkonen, Koski, Shinn, Wootten, & Sommerville, 2002). Treatments with MG were administered either as monocomponent baths or as multicomponent baths (in combination with formaldehyde and other products) (Sudova et al., 2007). However, particular concern is directed towards its use in the production of food fish: The high affinity and persistence of MG and, particularly, its reduced form (leucomalachite green—LMG) in animal tissues (Alborali, Sangiorgi, Leali, Guadagnini, & Sicura, 1997; Bauer, Dangschat, Knoppler, & Neudegger, 1988; Clifton‐Hadley & Alderman, 1987; Machova et al., 1996; Plakas, El‐Said, Stehly, Gingerich, & Allen, 1996) generates public health issues related to its potential carcinogenicity, teratogenicity and mutagenicity in humans, as suggested by experimental evidence in mammals (Culp et al., 2006; Mayer & Jorgenson, 1983; Werth, 1958). Following a considerable amount of studies demonstrating its toxicity and carcinogenicity in different animal species, the use of MG in the production of fish destined to human consumption is not authorized in the European Union (EU) (European Commission, 2010). However, since residues of MG and LMG have been detected in aquaculture products in monitoring programmes in EU Member States, the European Food Safety Authority (EFSA) established that food contaminated with MG/LMG at or below the reference point for action (RPA) of 2 μg/kg is unlikely to represent a public health concern (EFSA, 2016).

Several other chemicals have been used with different degrees of success for the treatment of *Saprolegnia* infections.

Among products specifically registered for aquaculture in some European countries, Pyceze® is employed in daily baths as prophylactic/therapeutic measure against fungal and bacterial infections in fish food eggs. The active ingredient of Pyceze® is bronopol (2‐bromo‐2‐nitropropane‐1,3‐diol), a broad‐range biocide used as a preservative in pharmaceutical and cosmetic industry (Bryce, Croshaw, Hall, Holland, & Lessel, 1978; Kumanova, Vassileva, Dobreva, Manova, & Kupenov, 1989; Toler, 1985), which would therefore pose no severe toxicological risks to human health. Nevertheless, its toxicity towards phytoplankton and zooplankton has been demonstrated. Furthermore, bronopol must be diluted prior to disposal, which significantly increases management costs (Nakagawa, Hara, Tokuyama, Takada, & Imamura, 2012).

Formalin, a solution of 37% formaldehyde, has been effectively employed to prevent (Bly, Quiniou, Lawson, & Clem, 1996; Schreier, Rach, & Howe, 1996) and treat (Cline & Post, 1972; Marking, Rach, & Schreier, 1994; Walser & Phelps, 1994) *Saprolegnia* infection in fish eggs. As a prophylactic measure, it inhibits cyst germination at a concentration of 250 mg/L (Bly et al., 1996). Daily flushes of formalin with 100, 200 and 400 mg/L increased per cent hatch of channel catfish *Ictalurus punctatus* eggs in comparison with non‐treated eggs (Walser & Phelps, 1994). Although formalin is still included in the list of allowed substances for all the food‐producing species and is marketed as a biocide for the disinfection of equipment and facilities (European Commission, 2010), it is not listed under the Biocidal Products Regulation (BPR) (European Parliament and Council, 2012), which is required for all products marketed as biocides. Moreover, formalin is currently not approved as a veterinary medicine for the treatment of live fish in most of the EU countries. However, in Spain, one formalin‐based product (Aquacen) has a marketing authorization for the control of *Philasterides dicentrarchi* in the farming of turbot *Psetta maxima* (Verner‐Jeffreys & Taylor, 2015). In the United States, formalin has been approved by Food and Drug Administration (USFDA, 2018) in three commercial formulations (Formalin‐F^™^, Formacide‐B and Parasite‐S®) for egg disinfection in aquaculture. From 1 January 2016, formaldehyde has been classified as a category 1B carcinogen (European Commission, 2014), therefore its use should be subjected to certain restrictions. Furthermore, besides the carcinogenic risk, formaldehyde represents a risk for exposed workers since it is a powerful irritant and allergenic substance.

Several other studies were performed to identify suitable compounds for the treatment or disinfection against *Saprolegnia*.

Ozone has shown effectiveness comparable to formalin in preventing saprolegniosis in brown trout (*Salmo trutta fario*) eggs (Forneris et al., 2003). The possible use of ozonized water in fish tanks is, however, controversial due to its strong oxidant properties (Fukunaga, Suzuki, & Takama, 1991) and potential toxicity to the branchial epithelium that could negatively affect respiration and osmoregulation.

Copper sulphate has been effectively used in aquaculture to control algal growth (James, Thomas, Gordon, & Frieda, 1998; Kiyoshi & Claude, 1993; Song, Marsh, Voice, & Long, 2011) and parasitic infection (Ling, Sin, & Lam, 1993; Schlenk, Gollon, & Griffin, 1998) proving effective against *Saprolegnia* both in vitro (Marking et al., 1994) and when administered to fish eggs in flow‐through systems (Straus, Mitchell, Carter, & Steeby, 2012). Sun, Hu, and Yang (2014) showed how copper sulphate inhibits the mycelium growth at concentrations ≥0.5 mg/L and the release of primary zoospores at 1 mg/L. On the other hand, sublethal concentrations can induce stress in rainbow trout *Oncorhynchus mykiss* which may become more susceptible to *Saprolegnia* (Carballo, Munoz, Cuellar, & Tarazona, 1995).

The fungicide activity of iodophores was demonstrated for the treatment of eggs in disinfectant baths, allowing to increase the hatching rate (Walser & Phelps, 1994). Despite their effectiveness and suitability for the disinfection of fish eggs, the potential use of iodophores for the treatment of a large number of fish is limited, due to the high concentrations needed (Bruno, van West, & Beakes, 2011) resulting in increased costs and potential toxicity.

Sodium chloride and a mixture of sodium and calcium chloride (26:1) tested as a prophylactic measure permitted to increase the hatching rate in salmonids; however, the required concentrations (20‐30 g/L) are hardly applicable in intensive farms (Edgell, Lawseth, Mclean, & Britton, 1993; Marking et al., 1994; Schreier et al., 1996; Waterstrat & Marking, 1995).

Hydrogen peroxide and boric acid are reported in the literature as promising compounds to control *Saprolegnia* infections. Hydrogen peroxide is an effective antimicrobial, showing antimycotic, antibacterial and antiviral activity coupled with low environmental impact, producing molecular oxygen and water as a result of its degradation. It was successfully tested against *Saprolegnia* mainly on fish eggs, resulting in increased hatching rates (Rach, Redman, Bast, & Gaikowski, 2005; Rach, Valentine, Schreier, Gaikowski, & Crawford, 2004; Small & Wolters, 2003; USFDA, 2007). At present, one hydrogen peroxide‐based product (Perox‐Aid®, Syndel, USA) is approved by the U.S. Food and Drug Administration (U.S. Food & Drug Administration (USFDA), 2018) for use in hatcheries.

Boric acid and borates have been used since the 19th century, as bactericides, fungicides and antiseptics (Quarles, 2001) and in several pesticides (EPA, 1993). This compound is safely used for the treatment of animal mycoses and in human medicine (De Seta, Schmidt, Vu, Essmann, & Larsen, 2009; Shi, Li, Qin, & Tian, 2011).

Ali, Thoen, Evensen, and Skaar (2014) tested boric acid in vitro against *Saprolegnia parasitica* and *Saprolegnia diclina* strains and in vivo on eggs and larvae of Atlantic salmon (*Salmo salar*). In vitro results showed inhibition of the mycelium growth at concentrations above 0.6 g/L, while in vivo tests allowed a high survival rate after continuous (0.2‐1.4 g/L) and intermittent (1‐4 g/L) exposure of eggs and larvae. These results suggest that boric acid could be safely used in aquaculture although its environmental impact must be carefully investigated.

The aim of this work was to perform an in vitro screening of promising molecules and commercial products against *Saprolegnia* spp. in order to provide information for the selection of safer and more environmentally friendly alternative treatment of saprolegniosis in aquaculture. The activity of new molecules is compared to the effectiveness of two compounds (malachite green, copper sulphate) used in the past to control saprolegniosis in aquaculture.

## 2 MATERIALS AND METHODS

### 2.1 Strains tested

Tests were carried out on three *Saprolegnia* strains: one reference strain of *S. parasitica* (CBS 223.65 provided by CSIC‐RJB Madrid, Spain) isolated in Holland from northern pike (*Esox lucius*), one field strain of *S. parasitica* (ITT 320/15/20) isolated in Italy from brown trout (*Salmo trutta fario*) and one field strain of *Saprolegnia delica* (ITT 290/15/15) isolated in Italy from rainbow trout (*Oncorhynchus mykiss*). Each strain has been tested in triplicate per each concentration.

### 2.2 Inocula

*Saprolegnia* spp. strains were maintained with periodic subcultures on glucose‐yeast (GY) agar medium (5 g D‐(+)‐glucose, 1 g yeast extract, 12 g agar in 1L deionized water) supplemented with 6 mg/L of penicillin and 10 mg/L of oxolinic acid (GY + P + Ox) (Alderman & Polglase, 1986) and kept at 18°C. For the in vitro trials, subcultures of the strains employed were incubated at 18°C until growth covered the full diameter of the dish (48‐72 hr). Inocula were obtained from the outer 10 mm of the culture, using a sterile 5‐mm‐diameter glass cannula (protocol I) or cutting a 4 × 4 mm piece with a sterile scalpel.

### 2.3 Products tested

Products under test include ten molecules (acetic acid, benzoic acid, boric acid, copper sulphate, iodoacetic acid, lactic acid, oxalic acid, tartaric acid, hydrogen peroxide and sodium percarbonate) belonging to different chemical classes and three commercial products (Actidrox®, De Marco, Italy; Detarox® AP, Perdomini, Italy; and Virkon^™^S, Dupont, UK). Malachite green was used as a reference compound, and copper sulphate was added because it is widely used to control saprolegniosis in aquacultured fish.

Each product was dissolved and/or diluted in sterile deionized water (with the exception of benzoic acid which was dissolved in absolute ethanol and subsequently diluted in water) at concentrations of 0.1; 1; 5; 10; 50; 100; 250; 500; 1,000; 5,000 ppm.

### 2.4 In vitro tests

Tests were performed following protocols I and II according to Alderman (1982).

For *protocol I*, different concentrations of the products under test were added to sterilized liquid GY agar at a temperature of 49°C. Mixtures were then distributed in six‐well plates (Ø 35 mm), allowing to test five different concentrations and one negative control. Following overnight solidification, a 5‐mm‐diameter well was excised in the centre of the agar using a sterile glass cannula. The well was then filled with a standard 5‐mm inoculum, culture surface uppermost. Plates were incubated at 18°C and checked after 24, 48, 72 hr and 6 days, determining the colony diameter of the growing mycelium as average of two axes measured at 90° from each other. The minimum inhibitory concentration (MIC) was defined as the lowest concentration inhibiting the growth of the mycelium after 6 days of incubation.

**Figure 1:**
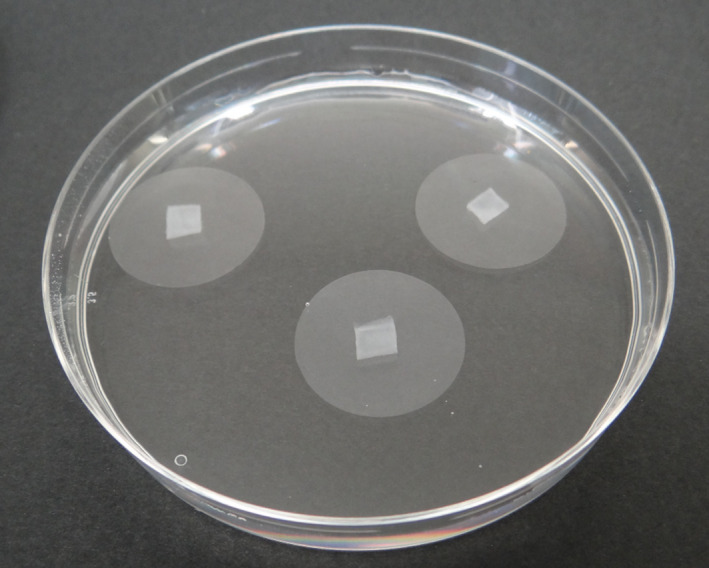
Protocol II: Filters supporting mycelium immersed in a solution of the product under test [Colour figure can be viewed at wileyonlinelibrary.com]

For *protocol II*, polycarbonate filter membranes (diameter 25 mm, pore size 5 μm—Whatman International Ltd., UK) were sterilized by autoclaving and then placed on the surface of 92‐mm petri dish (six per each petri dish). Filters were used as a support for the inoculum, obtained by placing a 4 × 4 mm piece of agar with growing mycelium. The inoculum was placed, inverted, at the centre of the filter. The dishes containing the filters were then incubated for 24 hr, until the resulting mycelial growth had almost reached the edge of the filters. The agar attached to the original inocula was then clipped off using hot forceps tips. The mycelia together with their supporting filters were lifted off the agar surface and placed in sterile 92‐mm petri dishes containing different concentrations of the products under test (Figure 1). Sterile deionized water was used as negative control. Filters were kept in contact with the solution for 1 hr and submitted to periodic agitation in order to achieve a better contact of the mycelium with the product under test. Subsequently, filters were washed twice with sterile water (for 5 and 30 min, respectively) and seeded on fresh dishes containing GY + P + Ox agar medium. Dishes were incubated at 18°C and checked after 24, 48, 72 hr and 6 days, determining the radial growth of the mycelium beyond the filter as average of two axes measured at 90° from each other. The minimum lethal concentration (MLC) was defined as the lowest concentration inhibiting any further growth of the mycelium after 6 days of incubation.

## 3 RESULTS

Minimum inhibitory concentrations and MLCs obtained for each tested compound and each strain are listed in Table 1. Triplicates were always consistent among each other.

**Table 1:** Minimum inhibitory concentrations (MIC) and minimum lethal concentrations (MLC) of tested compounds for the three *Saprolegnia* strains considered

Compound	MIC			MLC		
	A (ppm)	B (ppm)	C (ppm)	A (ppm)	B (ppm)	C (ppm)
Malachite green	5	5	5	5	5	5
Copper sulphate	250	250	250	1,000	5,000	5,000
Acetic acid	250	250	250	250	500	500
Benzoic acid	100	100	100	250	250	250
Boric acid	1,000	1,000	1,000	a	a	a
Iodoacetic acid	250	250	100	50	50	100
Lactic acid	500	500	5,000	500	500	1,000
Oxalic acid	500	500	1,000	1,000	5,000	5,000
Tartaric acid	500	500	1,000	a	a	a
Sodium percarbonate	a	a	a	a	a	a
Hydrogen peroxide	5,000	5,000	5,000	5,000	5,000	5,000
Actidrox®	5,000	5,000	5,000	500	500	500
Detarox®AP	1,000	1,000	1,000	100	100	100
Virkon^™^S	1,000	1,000	1,000	1,000	1,000	1,000

Notes

A, *Saprolegnia parasitica* CBS 223.65.

B, *Saprolegnia parasitica* ITT 320/15/20.

C, *Saprolegnia delica* ITT 290/15/15.

a Minimum inhibitory concentration or MLC not found at tested concentrations.

**Figure 2:**
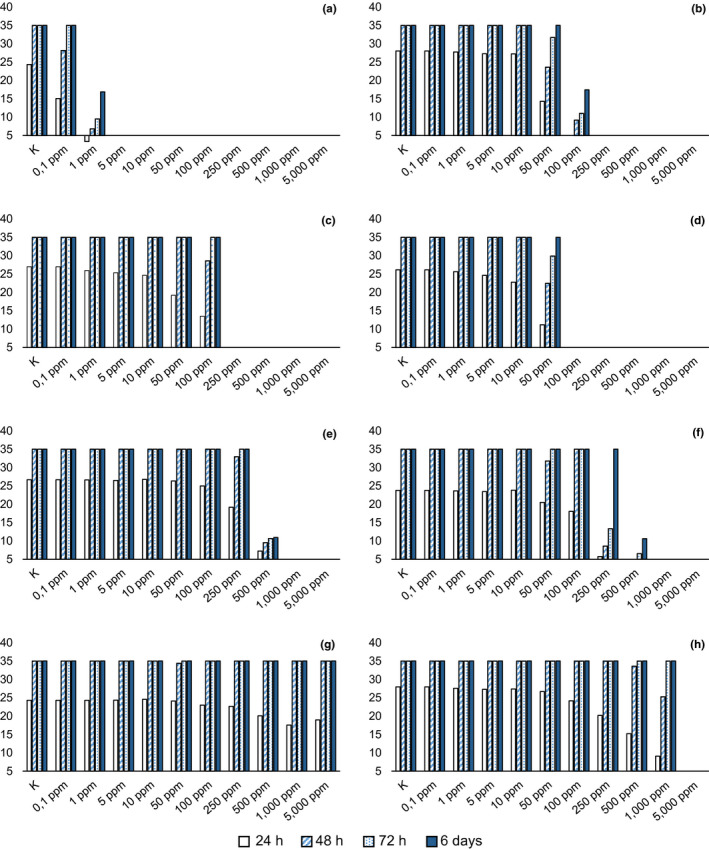
Average diameter (mm) of mycelium with different concentrations of compound in agar. (a) malachite green; (b) copper sulphate; (c) acetic acid; (d) benzoic acid; (e) boric acid; (f) oxalic acid; (g) sodium percarbonate; (h) hydrogen peroxide [Colour figure can be viewed at wileyonlinelibrary.com]

The effectiveness of malachite green (positive control) was confirmed in both tests (MIC = MLC = 5 ppm; Figure 2a). In agar, 50 ppm of copper sulphate was effective in slowing down the mycelium radial growth and in inhibiting the aerial mycelium at 6 days, but it produced complete inhibition only at 250 ppm (Figure 2b). In water trials, following 1‐hr exposure, copper sulphate had a lethal effect at concentrations of 1,000 ppm on *S. parasitica* strain CBS 223.65 and 5,000 ppm on the other two strains.

**Figure 3:**
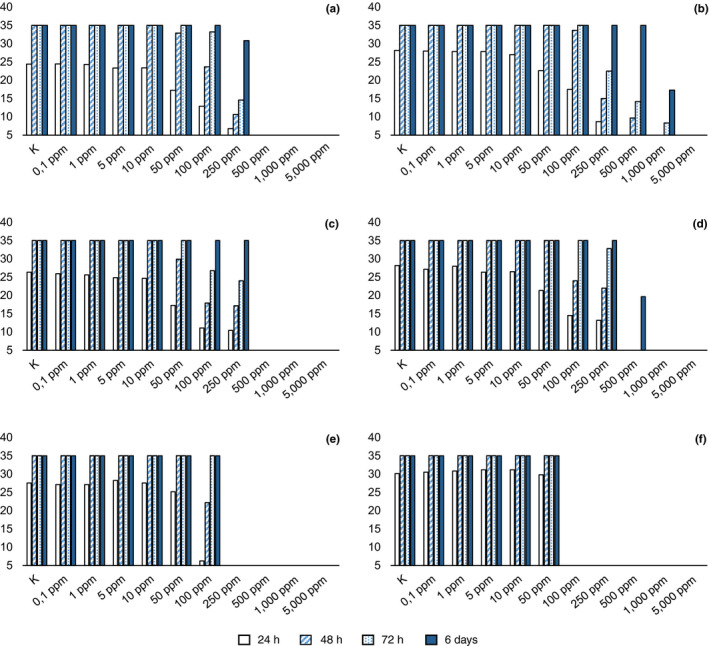
Average diameter (mm) of mycelium with different concentrations of product in agar. (a) Actidrox; (b) Detarox; (c): Virkon [Colour figure can be viewed at wileyonlinelibrary.com]

Among tested molecules, benzoic acid showed the lowest MIC (100 ppm), with radial mycelial growth considerably slowed down at 50 ppm (Figure 2d), followed by iodoacetic and acetic acid. With respect to iodoacetic acid, different MICs were observed in *S. parasitica* and *S. delica* (Figure 3e,f), but for all tested strains the inhibition of aerial mycelium on agar was observed at concentrations of 10 ppm at 6 days. Moreover, for *S. parasitica*, the MLC was lower than MIC.

For acetic acid, the MIC in agar resulted in 250 ppm (Figure 2c), but its activity in water was confirmed at 250 ppm only for *S. parasitica* strain CBS 223.65, while for the other two strains the MLC was set at 500 ppm.

Boric acid was effective in slowing down the mycelium growth in agar at 500 ppm and produced complete inhibition at 1,000 ppm (Figure 2e); MLC was not determined at tested concentrations.

For *S. parasitica*, the MIC of lactic acid was set at 500 ppm (Figure 3a) while for *S. delica* at 5,000 ppm (Figure 3b), but lower concentrations progressively reduced radial growth in agar. At 6 days, reduced growth was still noticeable at 250 ppm. The two *Saprolegnia* species tested showed a different behaviour with respect to lactic acid also in water trials and, in particular, MLC was lower than MIC for *S. delica*.

Tartaric acid completely inhibited the radial growth of *Saprolegnia* spp. for 72 hr at a concentration of 500 ppm, but some mycelial growth was observed for *S. delica* at 6 days; therefore, the MIC resulted in 1,000 ppm (Figure 3d), while for *S. parasitica* the MIC was confirmed at 500 ppm (Figure 3c). The same MICs were observed for oxalic acid, but a concentration of 250 ppm was effective in slowing down the mycelium growth for 72 hr, while a concentration of 500 ppm completely inhibited *Saprolegnia* growth for 48 hr (Figure 2f).

Minimum lethal concentration of tartaric acid was not determined at tested concentrations, whereas oxalic acid in water was lethal at 1,000 ppm for one *S. parasitica* strain (CBS 223.65) only, while the other two tested strains showed a MLC = 5,000 ppm.

Sodium percarbonate was effective only in slowing down the mycelium growth at the highest tested concentrations (5,000 ppm), but at 48 hr the diameter of mycelium was equal to the control (Figure 2g); therefore, MIC and also MLC were not determined at tested concentrations.

**Figure 4:**
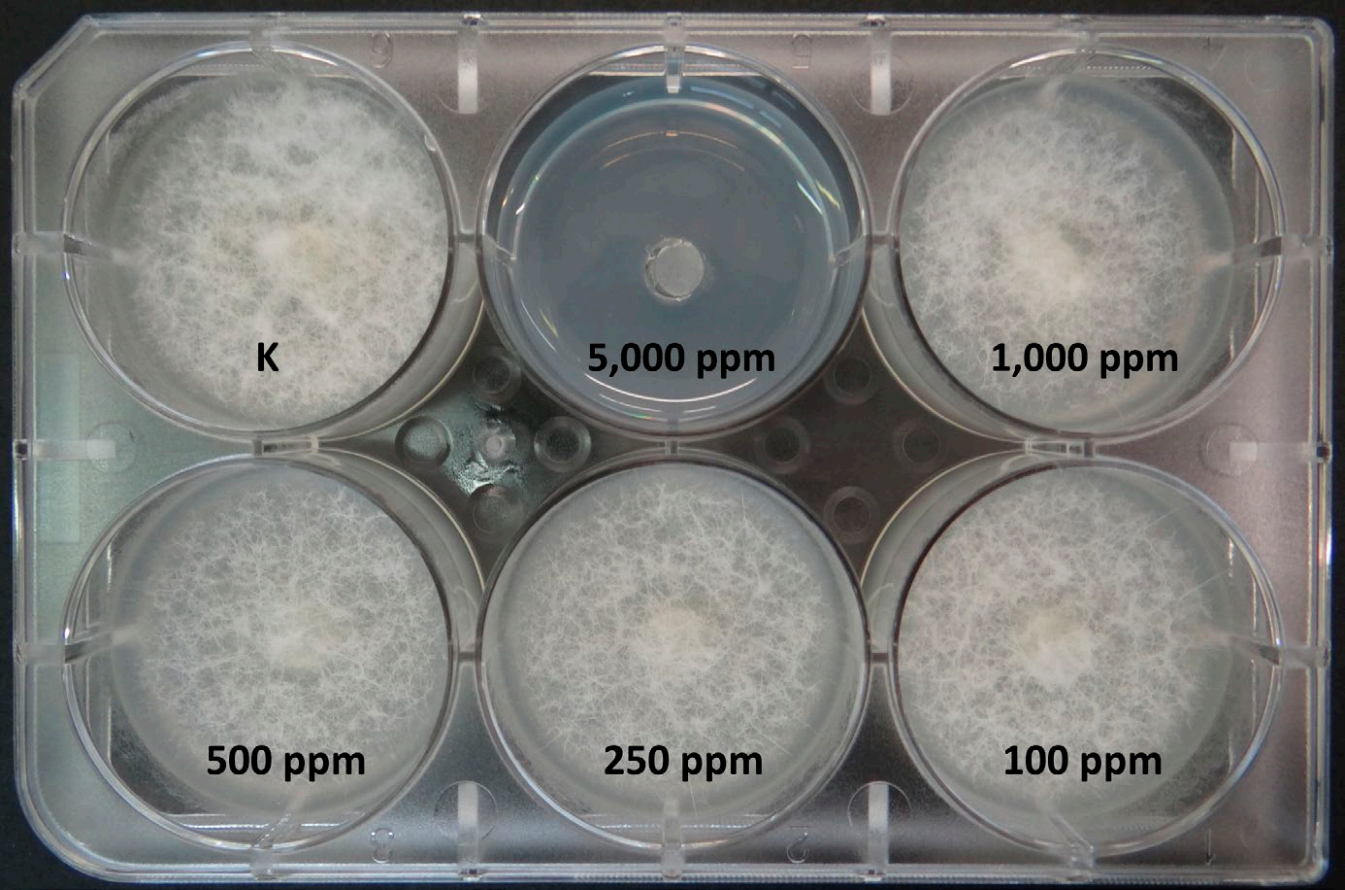
Mycelial growth after 6 days with different concentrations of hydrogen peroxide in agar, showing minimum inhibitory concentration at 5,000 ppm [Colour figure can be viewed at wileyonlinelibrary.com]

Hydrogen peroxide showed equal values of MIC and MLC (5,000 ppm) for all tested strains, but slower growth was observed at 250 ppm after 24 hr (Figure 2h; Figure 4).

**Figure 5:**
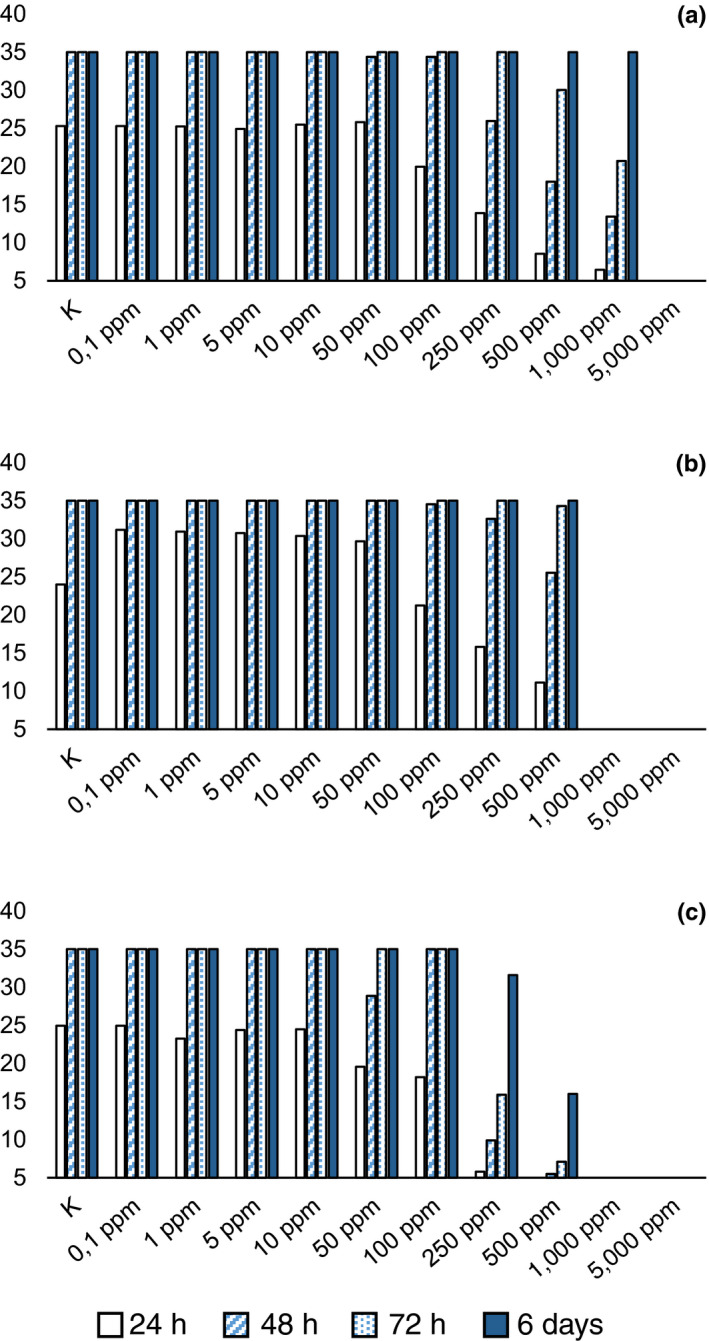
Average diameter (mm) of *Saprolegnia parasitica* and *Saprolegnia delica* mycelium with different concentrations of compound in agar. (a): *S. parasitica*, lactic acid; (b) *S. delica*, lactic acid; (c) *S. parasitica*, tartaric acid; (d) *S. delica*, tartaric acid; (e) *S. parasitica*, iodoacetic acid; (f) *S. delica*, iodoacetic acid [Colour figure can be viewed at wileyonlinelibrary.com]

As for commercial products, in agar, Actidrox® progressively slowed down mycelium growth with increasing concentrations of the product after 24 hr (Figure 5a); the MIC was set at 5,000 ppm. In water, this product showed a different effectiveness, resulting lethal at 500 ppm. Similarly, Detarox was 10‐fold more effective in water than in agar (MIC = 1,000 ppm ‐ Figure 5b; MLC = 100 ppm).

Finally, Virkon^™^S showed a MIC = MLC = 1,000 ppm (Figure 5c).

## 4 DISCUSSION

In our study, the activity of malachite green and its suitability as positive reference for the screening of antifungal compounds were confirmed (Bailey & Jeffrey, 1989; Marking et al., 1994). Although copper sulphate has been used in the past in fish culture for the control of parasitic and *Saprolegnia* infections (Straus et al., 2012; Sun et al., 2014), currently it is not approved for therapeutic use in aquaculture. According to recent research, the activity of this compound may vary according to different stages of *Saprolegnia* development: Sun et al. (2014) showed how copper sulphate prohibited the release of primary zoospores at concentrations ≥ 1 mg/L and inhibited mycelium growth at concentrations ≥ 0.5 mg/L for 24 hr. In our study, we evaluated the effect of copper sulphate on hyphal growth, showing that after 6 days of continuous exposure in agar, a complete inhibition was achieved only at 250 ppm (MIC); however, concentrations of 50 and 100 ppm inhibited the aerial mycelium. Also for iodoacetic acid, an inhibition of aerial mycelium on agar was observed at concentrations lower than MIC. The mechanisms that determine this would require further investigation, but chemically induced morphological hyphae changes with inhibition of aerial mycelium of *Saprolegnia* were already hypothesized in a previous work (Kaminskyj & Heath, 1992).

Sodium percarbonate is an environmentally safe compound that has been successfully tested in vitro against developmental stages of *Ichthyophthirius multifiliis* (Buchmann, Jensen, & Kruse, 2003; Heinecke & Buchmann, 2009); dosages of 12.5 mg/L for 180 min and 62.5 mg/L for 90 min were effective in killing *I. multifiliis* theronts (Buchmann et al., 2003), while tomont stage appears considerably more tolerant to the chemical. In our study, it was not possible to identify a MIC and MLC values of sodium percarbonate for *Saprolegnia* mycelium. However, a possible higher activity of this compound against other developmental stages of *Saprolegnia* (i.e., zoospores) cannot be excluded.

The compounds tested in the present study, with few exceptions (hydrogen peroxide, malachite green, Virkon^™^S), performed differently in protocols I and II (Table 1). As a general rule, the MLC was higher than the MIC, and for some of the tested molecules (boric and tartaric acid, sodium percarbonate) it was not possible to determine a MLC at tested concentrations. In water trials, *Saprolegnia* strains were kept in contact with the tested compounds for 1 hr only, while in agar such contact was continuous. Our results would therefore suggest that for some compounds, a more prolonged bath would possibly be required in order to achieve a lethal effect at lower concentrations.

However, one molecule (iodoacetic acid) and two commercial products (Actidrox®, Detarox®AP) resulted considerably more effective in water.

For Actidrox®, the different performances observed in the two protocols are possibly linked to the characteristics of the product itself, which releases peracetic acid (effective against a wide range of microorganisms) when solubilized in water. A similar behaviour was observed for Detarox®AP, an acidic sanitizer with oxidant properties, formulated with stabilized peracetic acid and hydrogen peroxide, that is widely used in the food industry for the disinfection of production equipment. Also for Detarox®AP, active compounds showed greater effectiveness in the presence of water. Both peracetic acid and hydrogen peroxide show a wide‐range antimicrobial activity (Baldry, 1983; Jussila, Makkonen, & Kokko, 2011; Kitis, 2004) and low environmental impact, and are considered suitable alternative sanitizers (Pedersen, Meinelt, & Straus, 2013). Particularly, results of in vitro assessments (Jaafar, Kuhn, Chettri, & Buchmann, 2013; Jussila et al., 2011; Picón‐Camacho, Marcos‐Lopez, Beljean, Debeaume, & Shinn, 2012; Picón‐Camacho, Marcos‐Lopez, Bron, & Shinn, 2012) highlight a promising role of peracetic acid‐based products for the control of parasitic and “oomycotic” infections (i.e., white spot disease, crayfish plague) in aquaculture. In water, Detarox®AP is degraded quickly, leaving residues of acetic acid and its salts; this would suggest a possible low environmental impact of the product but represent a challenge in controlling the effectiveness of the product in the farm. Peracetic acid decay can be significantly affected by organic matter content (Pedersen et al., 2013) and possibly influenced by other water properties (hardness, ion composition). Similarly, information available in the literature (Barnes, Gabel, Durben, Hightower, & Berger, 2004) suggests that possible differences in the activity of hydrogen peroxide may occur, depending on physical and chemical properties of the water.

Hydrogen peroxide alone is regarded as one of the most promising antibacterial (Wagner, Oplinger, Arndt, Forest, & Bartley, 2010), antiparasitic (Grave, Horsberg, Lunestad, & Litleskare, 2004; Picón‐Camacho, Marcos‐Lopez, Bron et al., 2012) and antifungal (Marking et al., 1994) compounds to be used in fish culture. MIC and MLC values obtained in the present study for this compound (5,000 ppm) would possibly limit its applicability in the field due to its toxicity on eggs at concentration > 1,000 μl/L (Gaikowski, Rach, Olson, Ramsay, & Wolgamood, 1998; Gaikowski, Rach, & Ramsay, 1999); however, after 24 hr at 500 ppm hyphal growth was considerably slowed down. Daily administration of the compound would therefore allow to effectively control the infection at a concentration lower than the observed MIC/MLC. These results confirm previous in vivo experiments conducted on eggs of rainbow trout (Marking et al., 1994; Schreier et al., 1996) and of chinook salmon *Oncorhynchus tshawytscha* (Waterstrat & Marking, 1995) that showed how concentrations of hydrogen peroxide ranging from 500 to 1,000 ppm are effective in controlling *S. parasitica* and *S. ferax* infection. Rach, Gaikowski, Howe, and Schreier (1998) and Rach et al. (2005) demonstrated that the toxicity of hydrogen peroxide to fish eggs varies according to different species, but was always above 1,000 μl/L; their results are in accordance with previous studies (Marking et al., 1994; Schreier et al., 1996) in documenting increased hatching rates in fish eggs treated with 1,000 ppm hydrogen peroxide.

Iodoacetic acid performed differently for *S. parasitica* and *S. delica* in the two protocols, being more effective at inhibiting *S. delica* in agar, but more lethal to *S. parasitica* in water. This product is toxic and corrosive but not considered hazardous to the environment (iodoacetic acid Sigma‐Aldrich Safety Data Sheet, https://www.sigmaaldrich.com/MSDS/MSDS/DisplayMSDSPage.do?country=IT&language=EN-generic&productNumber=I4386&brand=-SIAL&PageToGoToURL=https%3A%2F%2Fwww.sigmaaldrich.com%2Fcatalog%2Fproduct%2Fsial%2Fi4386%3Flang%3Dit)). It is included among the disinfection by‐products (DBPs) that could be produced in raw water after disinfection process. Furthermore, studies are in progress to evaluate whether subtoxic doses in water could represent a carcinogenic risk for humans (Marsà, Cortés, Hernándeza, & Marcosa, 2018).

Benzoic acid is an antifungal compound naturally produced in fruit to fight fungal infections (Brown & Swinburne, 1971); this molecule and its derivatives have long been used as antimicrobial preservatives in the food industry and as antifungal agents in topical preparations for the treatment of human infections (Rowe, Sheskey, & Quinn, 2009) and show activity at different levels of fungal development (Amborabé, Fleurat‐Lessard, Chollet, & Roblin, 2002). In our screening, benzoic acid has shown the lowest MIC (100 ppm). This compound is slightly soluble in water (<1 mg/ml at 20°C); therefore, in order to obtain the desired concentrations, it was first dissolved in absolute ethanol and then diluted with deionized water. Ethanol used for the solubilization of benzoic acid was tested in parallel, showing no effectiveness. This result is consistent with other evidence in the literature (Hoskonen, Heikkinen, Eskelinen, & Pirhonen, 2015).

With respect to protocol I, boric acid considerably slowed down the mycelium growth at 500 ppm but complete inhibition was achieved at 1,000 ppm, suggesting that the MIC of boric acid could be set in between the two concentrations (500 and 1,000 ppm), and be possibly close to values reported in previous investigations (600 ppm, Ali et al., 2014).

Virkon^™^S is a mixture of peroxygens, surfactants, organic acids and inorganic salts. Used in the farming industry for the disinfection of equipment and facilities, the product is described as effective against a wide range of viruses, bacteria and fungi (Virkon^™^S product container label). In aquaculture, Virkon^™^S is used at a concentration of 1% w/v for the disinfection of ponds and farm equipment (Sudova et al., 2007). Particularly, in salmonids, the experimental exposure to 1% Virkon^™^S for 15 min was effective in controlling *Gyrodactylus salaris* infection (Koski, Anttila, & Kuusela, 2016). However, to the best of our knowledge, the effectiveness of this product against *Saprolegnia* has never been tested. Our results suggest a possible use of Virkon^™^S at concentrations lower than 1% w/v for the control of saprolegniosis.

For some compounds (lactic acid, oxalic acid, tartaric acid), remarkably different behaviours were observed between the two *Saprolegnia* species here tested (*S. delica* and *S. parasitica*) during in vitro trials, in which higher concentrations were needed to inhibit *S. delica*. This oomycete species is widely distributed in natural freshwater systems (Sarowar, Van Den Berg, McLaggan, Young, & Van West, 2014), while *S. parasitica* is considered more primarily pathogenic (Van West, 2006). Information about the selective activity of antifungal compounds towards different *Saprolegnia* species would allow for the identification of compounds that inhibit the growth of the pathogenic *S. parasitica* but result harmless or less harmful to the many saprophytic species of *Saprolegnia* naturally occurring in freshwater ecosystems, thus helping to preserve the structure and diversity of natural oomycete communities.

In conclusion, the in vitro tests performed here show that, with the exception of sodium percarbonate, the compounds/products tested are effective against *Saprolegnia* spp., although at different concentrations. Among these, benzoic acid and iodoacetic acid showed the lowest MIC/MLC, respectively; however, acetic acid and peracetic acid‐based products, particularly in combination with hydrogen peroxide, represent promising candidates for controlling saprolegniosis in aquaculture, due to their effectiveness associated with low environmental impact.

In order to assess the possible field application of these most promising compounds, further tests will be necessary to evaluate their efficacy on different developmental stages of *Saprolegnia* (i.e., zoospores), their possible cytotoxic effects, and ultimately their safety and efficacy in vivo.

## CONFLICT OF INTEREST

The authors declare that there is no conflict of interest.
